# Microscopic risk assessment of highway weaving sections based on large-scale UAV trajectory data: What kinematic and interaction measures each detect

**DOI:** 10.1371/journal.pone.0361559

**Published:** 2026-11-05

**Authors:** Zhenghua Huang, Zhibing Yuan, Mengyuan Wang, Peng Fu

**Affiliations:** 1 School of Foreign Studies, Yiwu Industrial & Commercial College, Jinhua, China; 2 School of Industrial Affiliate, Xiangyang Auto Vocational Technical College, Xiangyang, China; Henan Polytechnic University, CHINA

## Abstract

Microscopic risk assessment of highway weaving sections relies largely on single-vehicle kinematic indicators, yet what such indicators actually detect is rarely tested against a measure of vehicle-to-vehicle interaction. This study analyses 12,256 complete vehicle trajectories collected by UAV at seven highway weaving sections, and evaluates every trajectory along two independent dimensions: a kinematics-based composite indicator combining longitudinal acceleration, lateral acceleration and longitudinal jerk with entropy-derived weights, and an interaction-based measure derived from time-to-collision with the leading vehicle. Because only seven sections are available, all section-level results are assessed by exact permutation as well as by parametric tests. Three findings follow. First, cumulative risk, the time integral of instantaneous risk, is mechanically dominated by traversal time (r = 0.989 with duration, r = 0.948 with weaving length at section level) and is unsuitable for comparing sections of differing length; time-averaged risk intensity is used instead. Second, individual behaviour dominates risk variation and both dimensions agree about it: merging and diverging movements carry higher risk and more conflicts than through movements, and heavy vehicles fewer conflicts than passenger cars (1.1% against 2.7% of trajectories below TTC = 3 s). Higher entry speed shortens exposure by 30.9% and lowers cumulative risk by 25.4% while raising risk intensity by 12.7%, so it reduces exposure rather than severity. Third, and centrally, the two dimensions respond differently to section configuration: task complexity is more strongly associated with interaction-based conflict rate than with kinematic risk intensity (permutation p = 0.028), although with seven sections neither association is significant on its own (p = 0.052 and p = 0.915), and the negative association between weaving length and risk intensity is directionally consistent across three methods but does not reach significance under permutation (p = 0.083). Kinematic indicators therefore characterise the demand placed on the individual driver, and no association between them and the interaction burden a section imposes was detected in these data; the two families of measure must be reported together. Risk tiers built on either kinematic feature are nonetheless externally valid, with conflict rate rising monotonically from 1.2% to 8.3% across intensity tiers.

## 1. Introduction

Highway weaving sections are critical segments connecting ramps and mainlines, where vehicles must complete merging and diverging manoeuvres within limited space, creating complex vehicle interaction patterns [1]. Because lane changes must be completed within short distances, collision risk in weaving sections is substantially higher than on ordinary segments, with accident rates reported at approximately two to three times those of ordinary highway segments [2]. As highway networks expand, the number of weaving sections continues to increase [3], and understanding how geometric configuration shapes risk in them is of considerable practical importance.

Research on weaving-section risk has moved from macroscopic capacity models [4–6] towards microscopic analysis. With the development of traffic conflict techniques, researchers adopted surrogate safety measures such as time-to-collision [7] and lane-change risk indices [8] to identify the spatiotemporal distribution of high-risk behaviour [9]. In parallel, the increasing accessibility of trajectory data [10,11] and the maturity of UAV and roadside video extraction [12,13] have made it possible to follow complete vehicle movements through a weaving section, and recent work on expressway diverging zones has extracted conflicts directly from UAV trajectories to model their causes and their real-time prediction [14,15].

Three methodological issues recur. The first concerns the nested structure of trajectory datasets: thousands of trajectories are collected from a small number of sections, so trajectories within a section share identical configuration parameters and are not independent [16], and ordinary regression underestimates standard errors for section-level predictors [17,18]. The effective sample for any section-level claim is therefore the number of sections, which in most such studies is under ten, and asymptotic p-values are unreliable at that size however many trajectories were collected.

The second concerns what a risk indicator measures. Kinematics-based indicators derived from a single vehicle’s acceleration profile are attractive because they can be computed at scale without identifying interacting pairs, but they cannot by construction observe how close a vehicle came to another. Whether they track conflict risk or driving smoothness is an empirical question [19], and answering it requires applying both families of measure to the same trajectories.

The third concerns the outcome measure itself. Cumulative risk, the time integral of instantaneous risk over a traversal, is widely used because it summarises total exposure. But an integral over time necessarily grows with the time taken, and traversal time is largely determined by segment length. Comparing cumulative risk across sections of differing length therefore mixes a substantive effect with an arithmetic one.

This study addresses all three. Using 12,256 complete trajectories from seven weaving sections recorded by UAV, we construct a kinematics-based composite risk indicator and, for the same trajectories, an interaction-based conflict measure, and analyse both within a two-level framework with exact permutation inference at section level. Three contributions follow. Cumulative risk, the time integral of Eq (10), turns out to be confounded by segment length, and we use the time-averaged intensity of Eq (12) in its place. The two families of measure differ in what they detect: task complexity registers more strongly on the interaction measure than on kinematic risk intensity. And a percentile-based risk tier system, validated against conflict rate, carries interaction-relevant meaning at trajectory level even though the indicator behind it does not register task complexity.

## 2. Data and methods

### 2.1 Data collection and preprocessing

Seven highway weaving sections were selected, covering a range of geometric configurations and traffic conditions. Data were collected by UAV at an altitude of 500 m during weekday morning peak periods (08:00–10:00) under clear, windless conditions. Table 1 summarises the configuration of each section. Weaving complexity is the sum of the diverging ratio and the merging ratio (Complexity = DR + MR), where the diverging ratio is the proportion of vehicles entering from the mainline that exit via the ramp and the merging ratio the proportion of vehicles exiting via the mainline that entered from the ramp. The lane configuration notation R-M-R denotes the number of lanes on the entry ramp, the mainline and the exit ramp respectively.

**Table 1:** Configuration of the seven weaving sections.

Section	Lane config. (R-M-R)	Length (m)	Design speed (km/h)	DR	MR	Complexity	Headway (s)
1	1-3-2	280	120	0.073	0.223	0.296	1.270
2	2-3-2	240	100	0.199	0.272	0.471	1.177
3	2-3-2	200	100	0.212	0.226	0.438	1.313
4	2-3-1	630	80	0.068	0.394	0.462	1.490
5	2-3-2	500	80	0.393	0.057	0.450	1.403
6	1-3-2	240	80	0.123	0.093	0.216	2.017
7	2-3-1	220	80	0.108	0.144	0.252	2.089

Ethics and permits. This study involved no human participants, no animals and no biological or environmental sampling, and approval from an institutional review board was therefore not required. No permit was required for access to the field sites: the weaving sections observed are public highway infrastructure, take-off and landing were carried out from publicly accessible ground beside the highway, no member of the research team entered the carriageway, and no restricted, protected or privately owned land was accessed. Data were collected in 2023. The unmanned aircraft was registered with the Civil Aviation Administration of China under its real-name registration scheme, and each flight was notified to that authority in advance through its Unmanned Aircraft System Operation Management platform. Under the procedure in force at the time, such notification constituted authorisation for flights of this class, and no approval reference number was issued. The recordings contain no licence plates, vehicle images or any other personal identifier. Only anonymous vehicle tracks and the kinematic quantities derived from them were retained, and no individual driver or vehicle can be identified from them.

Positional accuracy of the extraction was verified against known distances measured at the study sites: the mean absolute error was 0.06 m and the relative error 1.12%, which is adequate for microscopic traffic-flow analysis. Acceleration comes from the DataFromSky tracking output rather than from double differentiation of position.

Video processing and trajectory extraction were performed with DataFromSky. Videos were recorded at 30 fps, with eight videos at one site recorded at 48 fps, and trajectories were exported at every third video frame throughout, giving native sampling rates of 9.99 Hz for the 30 fps videos and 15.98 Hz for the 48 fps videos; material at two sites was additionally available at the full 29.97 Hz. To place all sections on an identical temporal footing, every trajectory was resampled onto a common 10 Hz grid, using integer decimation where the native rate is a multiple of 10 Hz and linear interpolation otherwise. All time derivatives were computed on the true time axis rather than on an assumed constant step.

Trajectories were retained only where the vehicle traversed the full weaving section, and each was truncated to the interval between its entry-gate and exit-gate crossing times, so that all derived quantities refer to the weaving section itself rather than to the camera field of view. Trajectory points with speeds above 150 km/h or below 20 km/h were removed, accounting for between 0.00% and 1.04% of points by section. Motorcycles and other non-car, non-goods-vehicle classes were excluded. A second-order Savitzky-Golay filter with a 0.5 s window was applied to the acceleration series before the jerk was derived [20].

A tracking-quality criterion was applied in addition. Automated tracking occasionally loses and reacquires a target, producing acceleration discontinuities that are not physically attainable. Trajectories in which longitudinal acceleration changed by more than 2 m/s² between consecutive 10 Hz samples — corresponding to an instantaneous jerk above 20 m/s³ — were excluded from analysis. This removed 199 trajectories (1.5%), distributed unevenly across sections (3, 13, 21, 5, 24, 71 and 62 for sections 1–7). They were kept in the traffic scene as potential leading vehicles for the interaction analysis and dropped only as analysis subjects. Of 13,302 tracked vehicles, 199 failed the tracking-quality criterion and 847 were ramp-to-ramp movements, leaving 12,256 trajectories for analysis. The full processed dataset for all seven sections is provided as Supporting Information (S2 Data), both as 10 Hz resampled time series and as trajectory-level features.

Path type is determined solely by the entry and exit gates recorded by DataFromSky: mainline entry to mainline exit is a through movement, ramp entry to mainline exit is a merging movement, and mainline entry to ramp exit is a diverging movement; ramp-to-ramp movements are excluded. Vehicle type follows the DataFromSky classification, with all goods-vehicle and bus categories grouped as heavy vehicles.

### 2.2 Kinematic risk indicators

The kinematic indicator quantifies risk from an individual vehicle’s motion, without requiring identification of interacting pairs. Three components are used: longitudinal acceleration, reflecting the intensity of speed change along the direction of travel; lateral acceleration, generated primarily during lane changes; and longitudinal jerk, reflecting the abruptness of driver input. Each is normalised and constrained to the unit interval:


$$\begin{array}{c} R_{\text{lon}}\left(t\right)=min\left(\frac{\left|a_{\text{lon}}\left(t\right)\right|}{a_{max}},\;1\right) \end{array}$$ (1)

$$\begin{array}{c} R_{\text{lat}}\left(t\right)=min\left(\frac{\left|a_{\text{lat}}\left(t\right)\right|}{a_{max}},\;1\right) \end{array}$$ (2)

$$\begin{array}{c} R_{\text{jerk}}\left(t\right)=min\left(\frac{\left|j\left(t\right)\right|}{j_{max}},\;1\right) \end{array}$$ (3)

$$\begin{array}{c} j\left(t\right)=\frac{\text{d}a_{\text{lon}}\left(t\right)}{\text{d}t} \end{array}$$ (4)

In Eqs (1) to (3), $a_{max}$ = 3.0 m/s² is applied to both acceleration components and $j_{max}$ = 2.5 m/s³. The upper bound is applied explicitly, so that each component and hence the composite indicator lie in [0,1] by construction. Both are normalising constants that fix the scale on which a component reaches its maximum, not thresholds of passenger comfort. The acceleration constant sits just below the 3.4 m/s² AASHTO treats as comfortably achievable by most drivers [21] and the 3.5 m/s² ceiling ISO 15622 places on automatic deceleration [22], and well above the 1.1 to 1.5 m/s² that ride-comfort research reports as acceptable in steady motion; it marks the upper bound of routine non-emergency manoeuvring. The jerk constant is the deceleration-jerk limit of ISO 15622 [22]. At these values 0.03% of longitudinal and 0.01% of lateral acceleration samples and 1.4% of jerk samples reach the bound.


$$\begin{array}{c} R\left(t\right)=w_{\text{1}}R_{\text{lon}}\left(t\right)+w_{\text{2}}R_{\text{lat}}\left(t\right)+w_{\text{3}}R_{\text{jerk}}\left(t\right) \end{array}$$ (5)

Weights are obtained by the entropy weight method, which assigns greater weight to components that discriminate more strongly among trajectories. Let $x_{ij}$ be the mean of component j over trajectory i, for m trajectories and three components. Each column is scaled to the unit interval and converted to a proportion, from which its information entropy and weight follow:


$$\begin{array}{c} {\tilde{x}}_{ij}=\frac{x_{ij}-{min}_{i}x_{ij}}{{max}_{i}x_{ij}-{min}_{i}x_{ij}} \end{array}$$ (6)

$$\begin{array}{c} p_{ij}=\frac{{\tilde{x}}_{ij}}{\sum_{i}{\tilde{x}}_{ij}} \end{array}$$ (7)

$$\begin{array}{c} E_{j}=-\frac{\text{1}}{lnm}\sum_{i=1}^{m}p_{ij}lnp_{ij} \end{array}$$ (8)

$$\begin{array}{c} w_{j}=\frac{1-E_{j}}{\sum_{k=1}^{\text{3}}(1-E_{k})} \end{array}$$ (9)

Applied to the trajectory-level component means this yields w_1_ = 0.344, w_2_ = 0.460 and w_3_ = 0.196. The weights describe how strongly each component discriminates within this sample, not its causal importance.

Four features are extracted from each risk time series, where T is the gate-to-gate traversal time and the integral is evaluated by the trapezoidal rule on the true timestamps:


$$\begin{array}{c} R_{\text{cum}}=\int_{\text{0}}^{T}R\left(t\right)\text{\textbackslash{}hspace\{0.17em}d\}t \end{array}$$ (10)

$$\begin{array}{c} R_{\text{peak}}={max}_{0\leq t\leq T}R\left(t\right) \end{array}$$ (11)

$$\begin{array}{c} R_{\text{int}}=\frac{R_{\text{cum}}}{T} \end{array}$$ (12)

$$\begin{array}{c} {\text{CV}}_{R}=\frac{\sigma_{R}}{\mu_{R}} \end{array}$$ (13)

where $\sigma_{R}$ and $\mu_{R}$ are the standard deviation and the mean of R(t) over the traversal. Cumulative risk measures total exposure and grows with traversal time; risk intensity measures risk per unit time and is independent of it.

### 2.3 Interaction-based conflict measures

Kinematic indicators cannot observe proximity to other vehicles. We therefore computed, for the same trajectories, measures based on time-to-collision with the leading vehicle. All vehicles present in a video were resampled onto the common 10 Hz grid, and trajectories were rotated into a road-aligned frame in which lateral offset was detrended against longitudinal position to remove road curvature. A leading vehicle is the nearest vehicle ahead whose lateral offset lies within ±1.8 m, approximately half a lane width; this identifies the relevant vehicle without requiring lane labelling, which is unstable in a weaving section where lane membership changes continuously. Writing s(t) for the longitudinal gap between the rear of the leading vehicle and the front of the following vehicle and Δv(t) for the closing speed, time-to-collision and its minimum over a traversal are


$$\begin{array}{c} \text{TTC}\left(t\right)=\frac{s\left(t\right)}{\Delta v\left(t\right)},\text{\textbackslash{}hspace\{1em}\}\Delta v\left(t\right)>0 \end{array}$$ (14)

$$\begin{array}{c} {\text{TTC}}_{min}={min}_{0\leq t\leq T}\text{TTC}\left(t\right) \end{array}$$ (15)

A trajectory is classified as experiencing a conflict when ${\text{TTC}}_{min}$ falls below a threshold τ, taken as 3 s with 1.5 s reported as a stricter alternative. We also record the time spent in conflict, following its use in recent conflict-risk work on expressway diverging zones [14]:


$$\begin{array}{c} \text{TSC}\left(\tau \right)=\int_{\text{0}}^{T}I\left[\text{TTC}\left(t\right)<\tau \right]\text{d}t \end{array}$$ (16)

where I[·] equals one when the condition holds and zero otherwise. Because time spent in conflict is itself a time integral we report it both raw and normalised by traversal time.

A valid minimum time-to-collision exists for 8,514 of the 12,256 trajectories (69.5%); the remainder never had an approaching leading vehicle within the section, and are treated as not having experienced a conflict. Conflict rate is reported both over all trajectories (the overall rate, used unless stated otherwise) and over those with a valid minimum time-to-collision (the conditional rate). A grid-based post-encroachment time was also tried. All traffic in a weaving section travels in one direction and lane-change angles are only a few degrees — the median heading difference between interacting pairs is 0.54° — so post-encroachment time on a spatial grid collapses into time headway and puts 73% of vehicles in conflict. Time-to-collision is used throughout.

### 2.4 Statistical framework

Primary inference operates at the trajectory level (n = 12,256). Differences between vehicle types are examined with Welch two-sample t-tests and differences among path types with one-way analysis of variance, with effect sizes reported as Cohen’s d and η². Because configuration parameters are shared by all trajectories within a section, trajectories are nested within sections; the intraclass correlation coefficient quantifies this clustering,


$$\begin{array}{c} \text{ICC}=\frac{\tau^{\text{2}}}{\tau^{\text{2}}+\sigma^{\text{2}}} \end{array}$$ (17)

where τ² is the between-section and σ² the within-section variance, both estimated from an intercept-only mixed model by restricted maximum likelihood. All models with section-level predictors are estimated as linear mixed-effects models with a random intercept per section,


$$\begin{array}{c} y_{ij}=\beta_{\text{0}}+\beta_{\text{1}}C_{j}+\gamma^{\prime }X_{ij}+u_{j}+\varepsilon_{ij},\;y_{ij}=\beta_{\text{0}}+\beta_{\text{1}}C_{j}+\gamma^{\prime }X_{ij}+u_{j}+\varepsilon_{ij} \end{array}$$ (18)

where i indexes trajectory and j section, $C_{j}$ is the standardised configuration parameter under test, ${𝐗}_{ij}$ the individual-level covariates (vehicle type, path type and entry speed) and $u_{j}$ the section random intercept. Individual-level effects are additionally estimated by ordinary least squares with section fixed effects.

Seven sections provide seven independent observations for any section-level claim, and asymptotic p-values are not trustworthy at that sample size. Every section-level association is therefore assessed by three methods: the parametric mixed-model p-value, an exact permutation test, and the simple correlation of the seven section means. The permutation test enumerates all 7! = 5,040 assignments of the seven observed configuration vectors to the seven sections. For each assignment the configuration variable is re-standardised and the model of Eq (18) is refitted by restricted maximum likelihood with the individual-level covariates unchanged; the p-value is the proportion of assignments, the observed one included, whose coefficient β_1_ is at least as large in absolute value as the observed one. To compare how two outcomes respond to the same configuration variable, each coefficient is divided by its permutation standard deviation and the difference is referred to its distribution over the same 5,040 assignments. We report all three methods and treat an association as established only where the permutation test supports it. Analyses were performed in Python using statsmodels; the significance level is α = 0.05.

## 3. Results

### 3.1 Risk indicator distributions and tier definitions

Table 2 summarises the distribution of the kinematic features. Cumulative risk shows the widest relative variability, reflecting the wide range of traversal times across sections of different length. Fig 1 shows the three distributions, with the tier cut-offs defined below marked as dashed lines on the two features used to define them.

**Table 2:** Descriptive statistics of risk features (n = 12,256).

Feature	Mean	SD	CV	P25	Median	P75	P95
Cumulative risk R_cum	2.219	0.867	0.391	1.605	2.002	2.658	3.903
Peak risk R_peak	0.300	0.081	0.272	0.244	0.291	0.344	0.446
Risk intensity R_int	0.131	0.037	0.281	0.105	0.125	0.151	0.200
Risk CV	0.459	0.115	0.250	0.379	0.457	0.535	0.649

**Fig 1:**
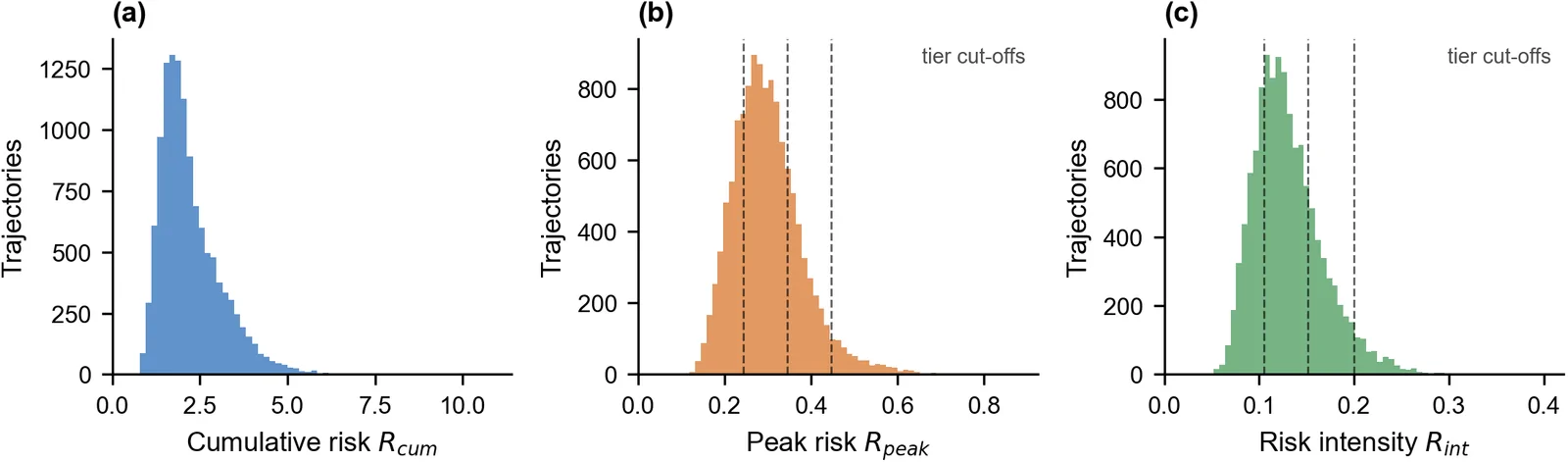
Distributions of the three kinematic risk features.

To make indicator values interpretable, trajectories are classified into four tiers by sample percentiles, following data-driven stratification practice in kinematic driving-risk studies [23]. Tiers are defined on the two features that do not grow with traversal time: risk intensity, used for section-level screening, and peak risk, used for behaviour-level screening. Thresholds are given in Table 3; they are relative to the observed sample rather than absolute safety standards. Fig 2 shows one representative trajectory from each intensity tier, chosen as the trajectory closest to its tier median within the indicated path type. Speed, longitudinal acceleration and composite instantaneous risk are plotted against time in the section, with the Very High threshold marked. The progression is visible in the acceleration traces: the Low and Medium examples fluctuate within ±1 m/s², whereas the two diverging examples sustain deceleration of more than 1 m/s² and cross the threshold repeatedly.

**Table 3:** Risk tier thresholds.

Tier	Risk intensity (section screening)	Peak risk (behaviour screening)	Share
Low	< 0.1048	< 0.2436	25%
Medium	0.1048–0.1510	0.2436–0.3442	50%
High	0.1510–0.1997	0.3442–0.4455	20%
Very High	> 0.1997	> 0.4455	5%

**Fig 2:**
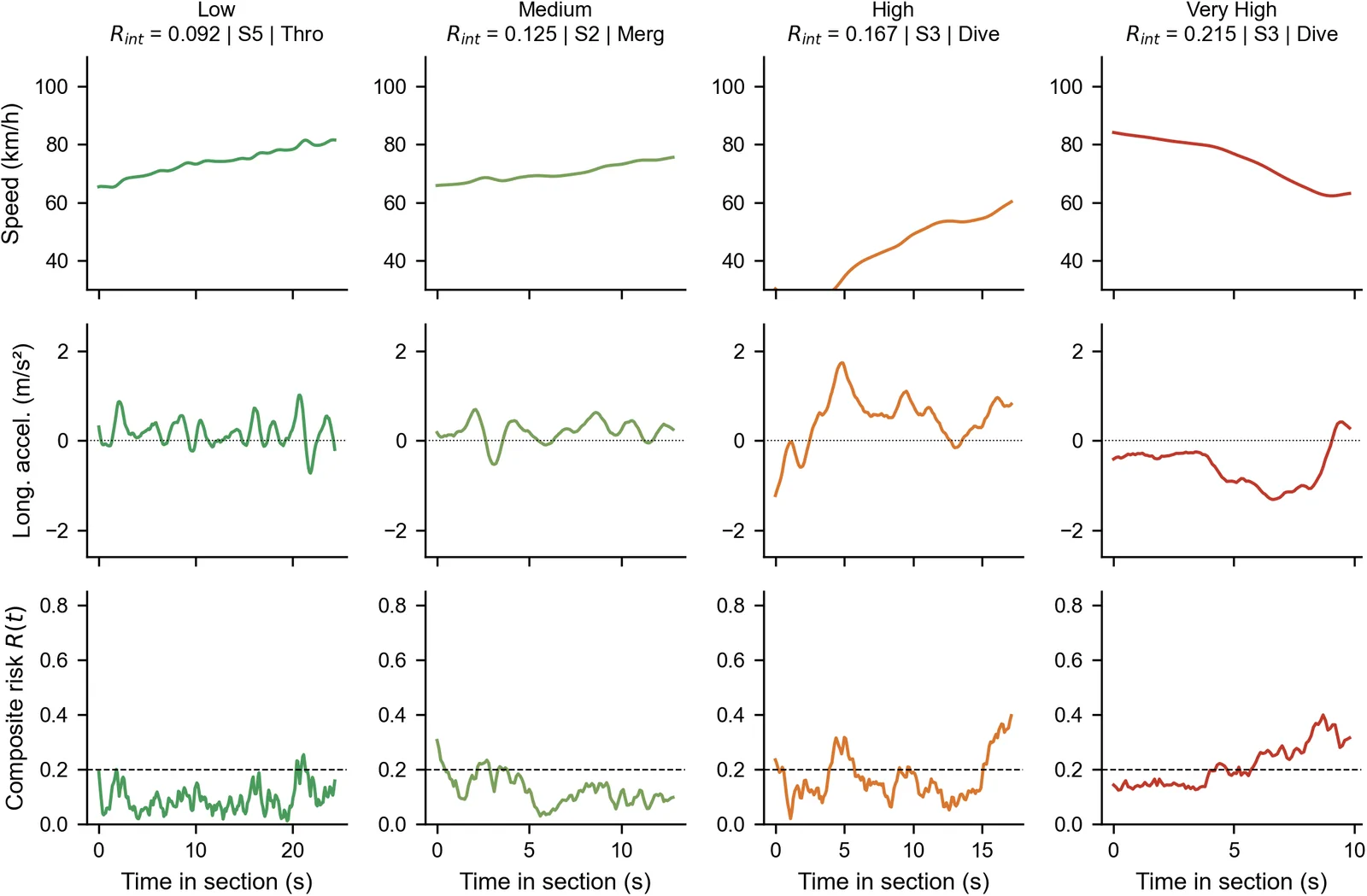
Representative trajectories from each risk-intensity tier.

### 3.2 Individual-level effects

Under the common normalising constants used throughout, heavy vehicles (n = 1,488) record lower kinematic risk than passenger cars (n = 10,768) on every feature (Table 4), and the difference persists after absorbing section characteristics (β = −0.160 on cumulative risk, −0.0124 on intensity, −0.0154 on peak risk, all p < 0.001). Component means indicate why: heavy vehicles record lower longitudinal acceleration (0.107 against 0.123) and lower jerk (0.223 against 0.236), with essentially identical lateral acceleration.

**Table 4:** Vehicle type effects, and their dependence on the normalising constants.

Constants	Feature	Car	Heavy vehicle	Δ (%)	Cohen’s d
Common (3.0, 2.5)	Cumulative risk	2.245	2.034	−9.4%	−0.244
	Risk intensity	0.132	0.124	−5.7%	−0.206
	Peak risk	0.302	0.289	−4.3%	−0.158
Type-specific (heavy 2.0, 1.5)	Cumulative risk	2.245	3.098	+38.0%	+0.956
	Risk intensity	0.132	0.189	+43.6%	+1.504
	Peak risk	0.302	0.419	+38.9%	+1.410

This comparison is not, however, identified by the kinematic indicator. Because each component is normalised by a constant, the relative standing of the two vehicle types depends entirely on the constants chosen. Adopting lower constants for heavy vehicles (2.0 m/s² and 1.5 m/s³ in place of 3.0 and 2.5) reverses the comparison completely, from −9.4% to +38.0% and from Cohen’s d = −0.244 to +0.956. Choosing between the two settings would require independent evidence on what counts as routine motion for each vehicle class. We keep the common constants for comparability and draw no conclusion about vehicle type from the kinematic indicator. Both settings in Table 4 are significant at p < 0.001; only the sign differs.

Path type is the strongest individual-level predictor. Merging and diverging movements carry markedly higher cumulative and peak risk than through movements (Table 5), and the difference persists after section fixed effects are included (merging β = +0.290 and diverging β = +0.433 on cumulative risk, both p < 0.001). For risk intensity the differences among path types are much smaller (η² = 0.002), because merging and diverging vehicles also take longer to traverse the section, which dilutes their time-averaged risk; the intensity effect is nonetheless significant once section is controlled. Fig 3 shows the corresponding distributions, vehicle type in the upper row and path type in the lower row.

**Table 5:** Path type effects (one-way ANOVA).

Feature	Through (n = 7,087)	Merging (n = 2,840)	Diverging (n = 2,329)	F	η²
Cumulative risk	1.934	2.611	2.610	1068.0	0.148
Peak risk	0.288	0.318	0.315	190.0	0.030
Risk intensity	0.1300	0.1340	0.1298	13.7	0.002

**Fig 3:**
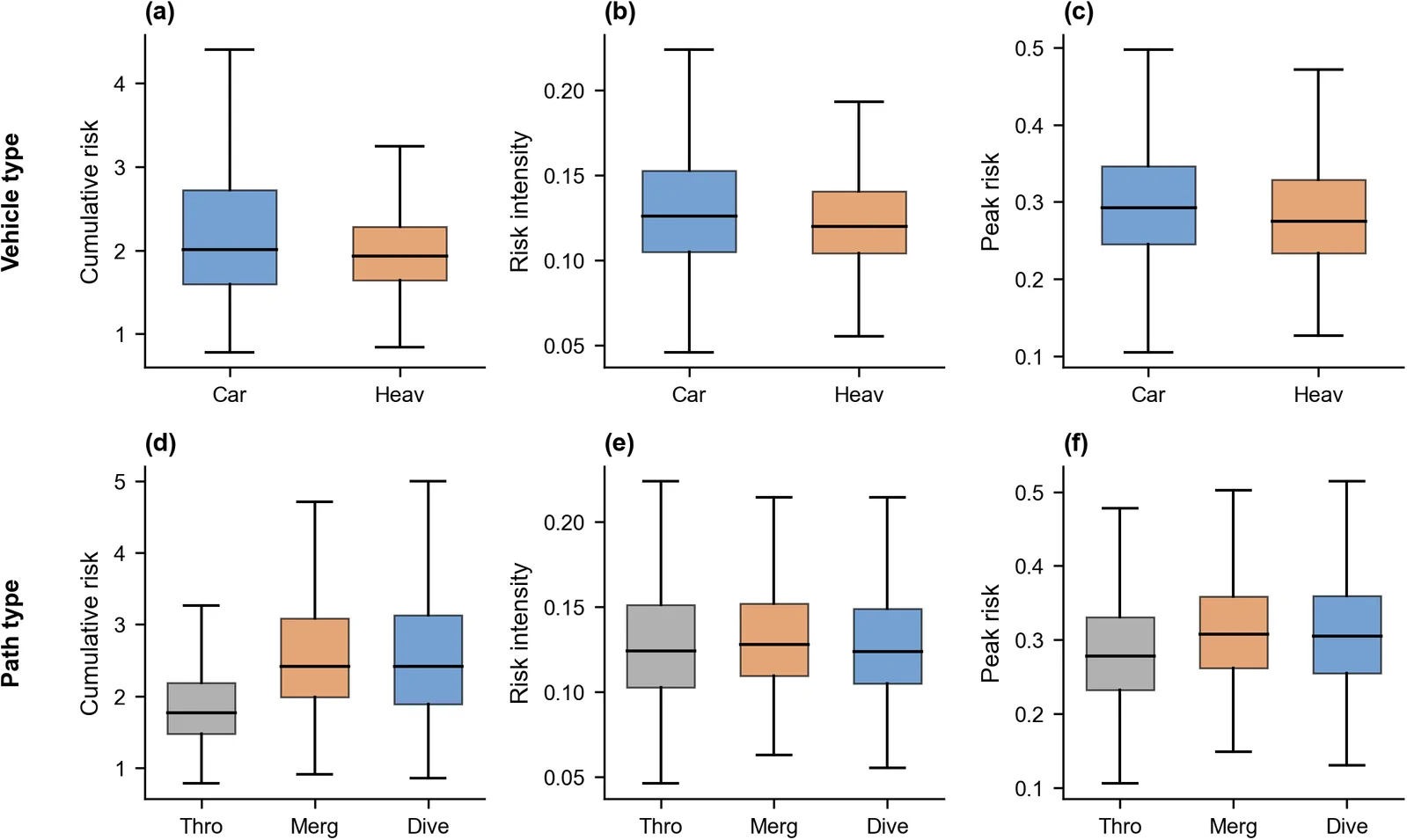
Individual-level effects of: vehicle type (a–c) and path type (d–f).

Entry speed illustrates most clearly why cumulative risk and risk intensity must be distinguished. Entry speed is negatively correlated with cumulative risk (r = −0.314) but positively correlated with risk intensity (r = +0.224), and essentially uncorrelated with peak risk (r = +0.011). The reason is exposure time: entry speed correlates with traversal time at r = −0.337, and traversal time correlates with cumulative risk at r = +0.781. Table 6 reports all three quantities across entry-speed tertiles. Vehicles entering fastest spend 30.9% less time in the section and accumulate 25.4% less total risk, but experience 12.7% more risk per unit time: entry speed shortens exposure while raising instantaneous demand. Fig 4 makes the divergence visible. Traversal time and cumulative risk fall monotonically with entry speed, whereas risk intensity is flat up to roughly 75 km/h and rises thereafter; points are individual trajectories and the line joins means of 5 km/h bins holding at least 30 trajectories.

**Table 6:** Entry speed tertiles: exposure and intensity reported separately.

Tertile	Entry speed (km/h)	Traversal time (s)	Cumulative risk	Risk intensity
Lower	47.6	20.49	2.537	0.1280
Middle	65.9	19.30	2.229	0.1203
Upper	82.0	14.16	1.892	0.1443
Upper vs lower	+72.1%	−30.9%	−25.4%	+12.7%

**Fig 4:**
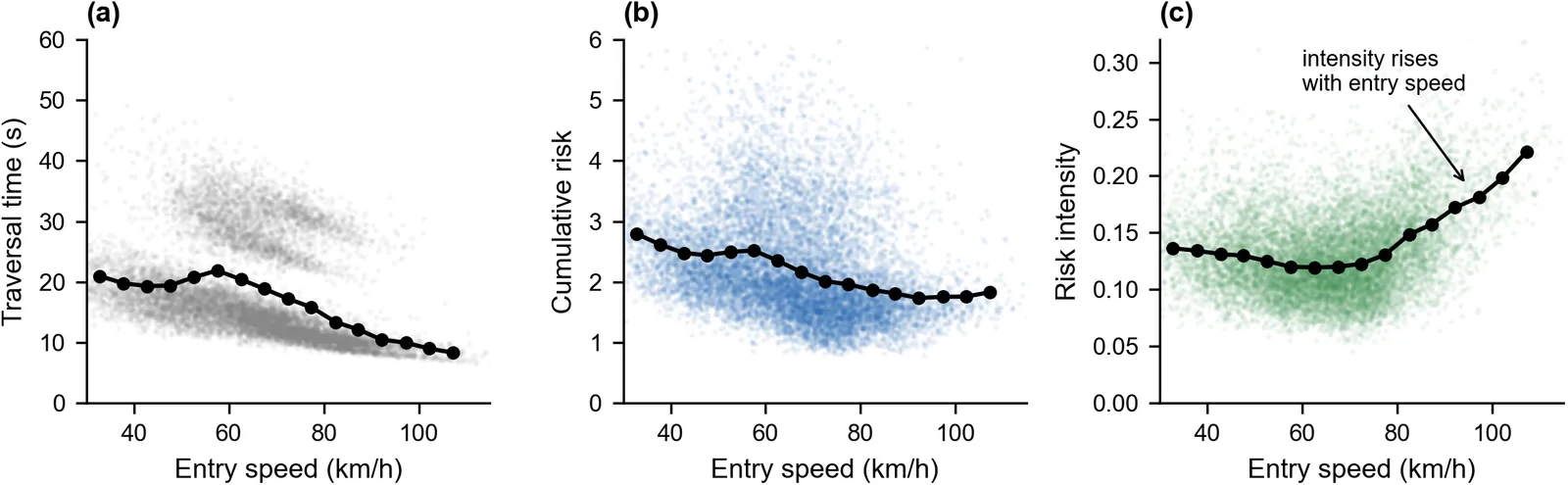
Entry speed against: (a) traversal time, (b) cumulative risk and (c) risk intensity.

Table 7 reports the multi-factor model with section fixed effects and heteroskedasticity-robust standard errors. Diverging path type carries the largest coefficient on cumulative risk, and the model explains 61.4% of its variance. The same predictors explain 41.3% of the variance in risk intensity but only 10.2% of the variance in peak risk, indicating that peak risk is governed by transient events these covariates do not capture. Section dummies are included in every model but not shown, and all coefficients in Table 7 are significant at p < 0.001.

**Table 7:** Individual-level predictors with section fixed effects (HC3 standard errors).

Predictor	Cumulative risk	Risk intensity	Peak risk
Heavy vehicle	−0.160***	−0.0124***	−0.0154***
Merging path	+0.290***	+0.0256***	+0.0440***
Diverging path	+0.433***	+0.0141***	+0.0399***
Entry speed (per km/h)	−0.0110***	+0.0010***	+0.0010***
R²	0.614	0.413	0.102

### 3.3 Section-level distributions

Table 8 reports section means. Traversal time varies from 11.1 s to 32.4 s and tracks weaving length almost exactly (r = 0.989). Cumulative risk follows the same pattern (r = 0.948 with length), whereas risk intensity moves in the opposite direction (r = −0.767). Section 4, the longest at 630 m, records the highest cumulative risk and the lowest intensity; section 3, the shortest at 200 m, records the reverse. This is the arithmetic relationship set out in the Introduction, and the reason cumulative risk is not used as a section-level outcome. Conflict rate in Table 8 is the proportion of trajectories whose minimum time-to-collision falls below 3 s, given over all trajectories and over those with a valid time-to-collision. Fig 5 plots the section means against weaving length, one point per section: traversal time and cumulative risk rise with length while risk intensity falls.

**Table 8:** Section-level means.

Section	n	Valid TTC, n (%)	Length (m)	Complexity	Traversal time (s)	Cumulative risk	Risk intensity	Peak risk	Conflict rate, overall	Conflict rate, conditional
1	2,358	1,732 (73.5%)	280	0.296	17.07	2.167	0.1278	0.299	1.53%	2.08%
2	2,206	1,598 (72.4%)	240	0.471	14.84	2.048	0.1416	0.302	3.13%	4.32%
3	2,347	1,564 (66.6%)	200	0.438	12.25	1.921	0.1624	0.321	3.62%	5.43%
4	1,429	1,147 (80.3%)	630	0.462	32.42	3.469	0.1074	0.325	3.36%	4.18%
5	1,563	1,229 (78.6%)	500	0.450	28.16	2.947	0.1039	0.289	2.43%	3.09%
6	1,206	674 (55.9%)	240	0.216	12.90	1.579	0.1234	0.275	1.41%	2.52%
7	1,147	570 (49.7%)	220	0.252	11.11	1.391	0.1259	0.264	0.96%	1.93%

**Fig 5:**
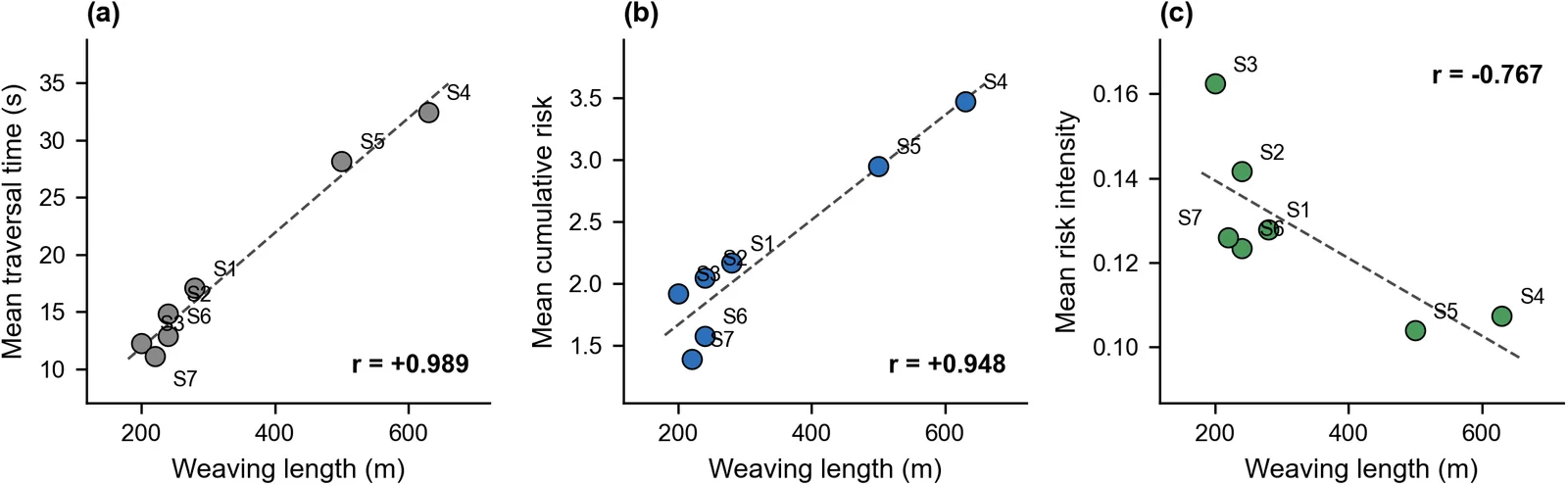
Section means against weaving length: (a) traversal time, (b) cumulative risk, (c) risk intensity.

### 3.4 What the kinematic dimension detects

The intraclass correlation coefficients (Table 9) confirm that clustering differs markedly among outcomes. Intervals come from a parametric bootstrap with 400 replications.

**Table 9:** Variance components and intraclass correlation coefficients (null models, REML).

Feature	τ² (between)	σ² (within)	ICC	95% CI
Cumulative risk	0.53865	0.37450	0.590	[0.288, 0.771]
Risk intensity	0.00040	0.00098	0.290	[0.076, 0.500]
Peak risk	0.00050	0.00628	0.074	[0.020, 0.146]

Table 10 reports each configuration parameter under all three inferential approaches. Only one association survives all of them, and it is the arithmetic one: weaving length predicts cumulative risk, because longer sections take longer to traverse. Every other kinematic association fails the permutation test. The negative association between weaving length and risk intensity — the substantively interesting one, and the direction the design literature would predict — is supported by the parametric model (p = 0.009) and by the section-mean correlation (r = −0.767, p = 0.044), but does not reach significance under permutation (p = 0.083). It is suggestive rather than established at this sample size. Task complexity and headway show no association with any kinematic outcome under permutation. Each row of Table 10 comes from a single-predictor mixed model with a random intercept per section and individual-level covariates; permutation p is computed over all 5,040 reassignments of the seven configuration vectors, and the section-mean correlation from the seven section means. Fig 6 presents the same coefficients from the joint model as a forest plot, with horizontal scales differing between panels because the outcomes are on different scales.

**Table 10:** Configuration parameters under three inferential approaches.

Outcome	Predictor	β	Parametric p	Permutation p	Section-mean r (p)
Cumulative risk	Weaving length	+0.5848	< 0.001	0.001	+0.948 (0.001)
	Task complexity	+0.3773	0.065	0.166	+0.684 (0.090)
	Headway	−0.2238	0.343	0.475	−0.477 (0.279)
Risk intensity	Weaving length	−0.0148	0.009	0.083	−0.767 (0.044)
	Task complexity	+0.0011	0.902	0.915	+0.064 (0.892)
	Headway	−0.0075	0.328	0.399	−0.266 (0.565)
Peak risk	Weaving length	+0.0051	0.553	0.602	+0.400 (0.374)
	Task complexity	+0.0120	0.084	0.169	+0.749 (0.053)
	Headway	−0.0150	0.004	0.061	−0.740 (0.057)

**Fig 6:**
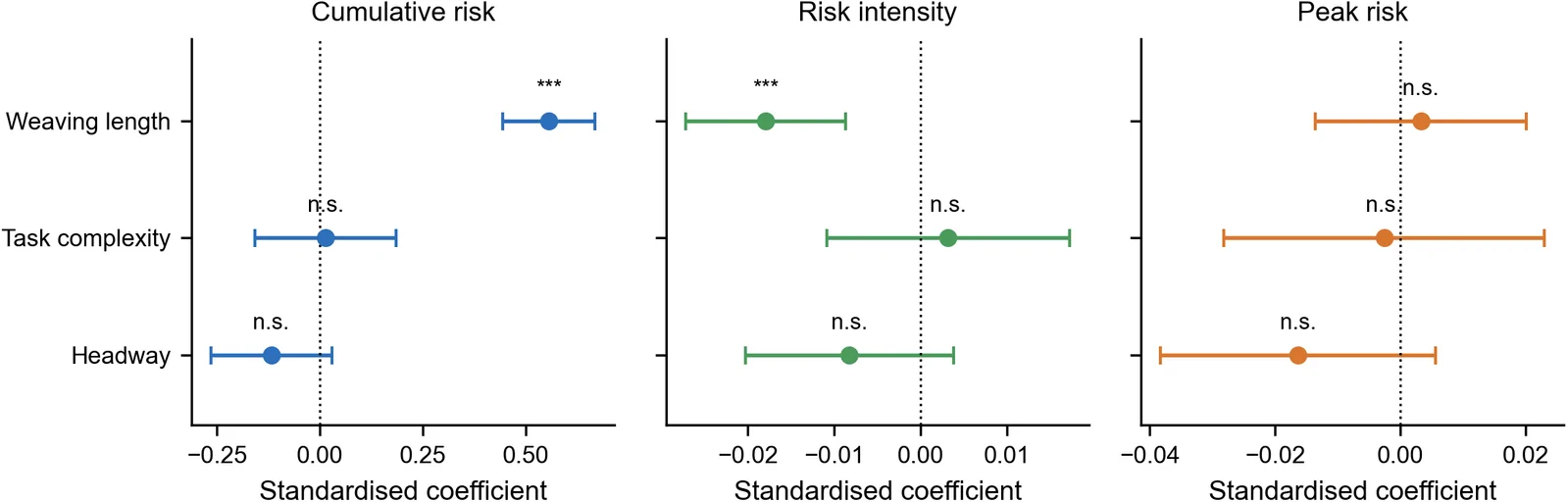
Mixed-effects coefficients for the three configuration parameters under each outcome.

Fitting all three configuration parameters jointly is not advisable here. Task complexity and headway correlate at −0.767 across the seven sections, so sections with more weaving activity also tend to have shorter headways; variance inflation factors are 1.50, 3.52 and 2.77 for length, complexity and headway. The joint model for cumulative risk fails to converge, and the models for the other two outcomes converge only after the optimiser falls back to an alternative algorithm, returning boundary solutions. Single-predictor models, reported above, converge without difficulty.

### 3.5 What the interaction dimension detects

The interaction measure behaves quite differently. Conflict rate varies from 0.96% to 3.62% across sections and tracks task complexity closely (r = +0.905, p = 0.005) while showing little relation to weaving length (r = +0.340, p = 0.456). The four sections with high task complexity (0.438 to 0.471) record conflict rates between 2.4% and 3.6%, while the three with low complexity (0.216 to 0.296) record between 0.96% and 1.53%, and this holds regardless of length: section 4, at 630 m, and section 3, at 200 m, record similar rates. The conditional rate shows the same pattern (r = +0.789 with complexity, p = 0.035). More complex sections also have a larger share of trajectories with a valid time-to-collision (r = +0.782, p = 0.038), as more weaving brings more leading vehicles to approach.

In the mixed model the association between task complexity and conflict rate is β = +0.0070, with permutation p = 0.052, where task complexity reaches p = 0.915 on the corresponding kinematic outcome. On the conditional rate the coefficient is almost unchanged (β = +0.0069), but with 8,514 rather than 12,256 trajectories the permutation p is 0.166. The association weakens at the stricter conflict threshold (TTC < 1.5 s: p = 0.217) and for time spent in conflict (p = 0.354). Table 11 reports the interaction outcomes under the same three inferential approaches used for the kinematic outcomes in Table 10, so the two can be compared row by row.

**Table 11:** Interaction-based outcomes under the same three inferential approaches.

Outcome	Predictor	β	Parametric p	Permutation p	Section-mean r (p)
Conflict rate (TTC < 3 s), overall	Weaving length	+0.00146	0.693	0.673	+0.340 (0.456)
	Task complexity	+0.00697	0.001	0.052	+0.905 (0.005)
	Headway	−0.00525	0.064	0.185	−0.696 (0.083)
Conflict rate (TTC < 3 s), conditional	Weaving length	−0.00084	0.868	0.831	+0.109 (0.817)
	Task complexity	+0.00692	0.064	0.166	+0.789 (0.035)
	Headway	−0.00416	0.305	0.423	−0.575 (0.177)
Conflict rate (TTC < 1.5 s), overall	Task complexity	+0.00235	0.096	0.217	+0.662 (0.106)
Time spent in conflict (rate)	Task complexity	+0.00029	0.232	0.354	+0.621 (0.137)

Because both coefficients are available for each of the 5,040 assignments, the difference between the two dimensions can be tested directly. Task complexity is more strongly associated with conflict rate than with risk intensity, by 1.81 permutation standard deviations (p = 0.028). The same comparison with peak risk is not significant (p = 0.477), nor is the reverse comparison for weaving length, which is more strongly associated with risk intensity than with conflict rate (p = 0.114). Fig 7 shows the contrast directly: conflict rate against complexity, conflict rate against length, and risk intensity against length.

**Fig 7:**
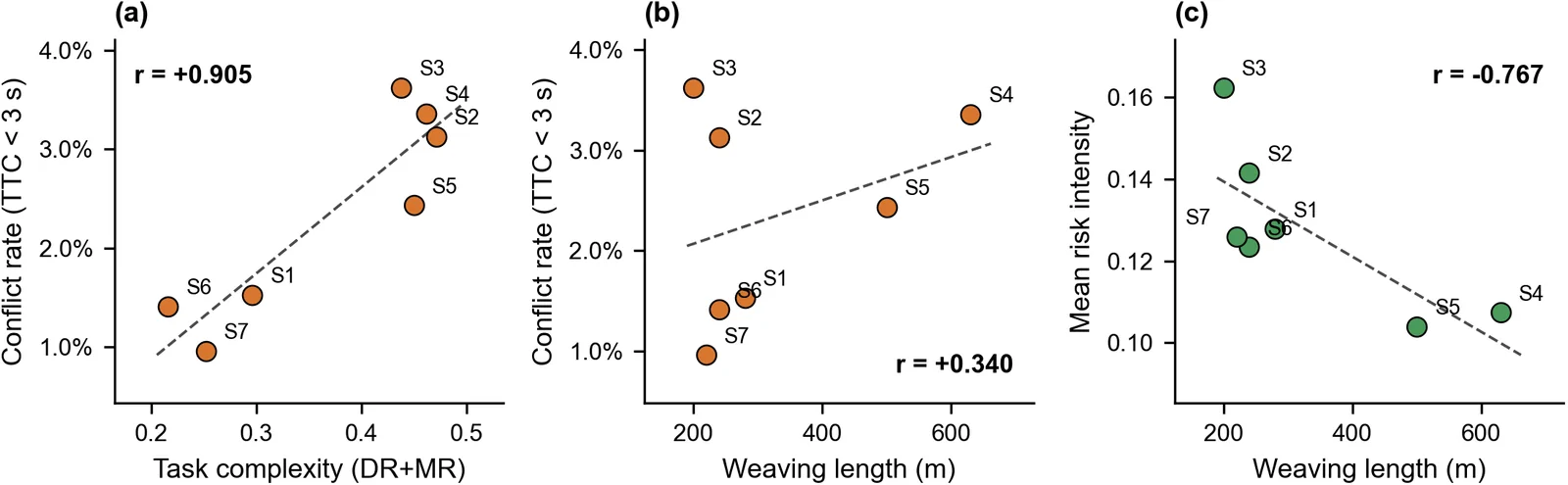
The two dimensions respond differently: (a) conflict rate against task complexity, (b) conflict rate against weaving length, (c) risk intensity against weaving length.

At the level of individual behaviour the two dimensions agree. In a mixed model with a random intercept per section, merging (β = +0.0100, p = 0.020) and diverging (β = +0.0114, p = 0.006) movements carry higher conflict probability than through movements, reproducing the kinematic ordering, and heavy vehicles carry lower conflict probability (β = −0.0195, p < 0.001). Overall conflict rates are 1.83% for through, 3.38% for merging and 3.35% for diverging movements, and 1.08% for heavy vehicles against 2.68% for passenger cars, with conditional rates in the same order (2.77%, 4.64% and 4.43%; 1.84% against 3.77%). Because the interaction measure involves no normalising constants, it settles the vehicle-type question that the kinematic indicator could not: heavy vehicles are involved in fewer close interactions at these sections. Time spent in conflict gives the same ordering, at 0.094%, 0.161% and 0.204% of traversal time for through, merging and diverging movements.

Time spent in conflict is worth a closer look. Conflicts are rare — 2.5% of trajectories record any time below TTC = 3 s, with a mean duration of 0.87 s among those that do — so the measure accumulates according to how often conflicts occur rather than how long a vehicle remains in the section. Its correlation with weaving length is + 0.456 raw and −0.295 once normalised by traversal time, against +0.948 for cumulative kinematic risk. Time-integrated measures are therefore not uniformly confounded by segment length; what matters is whether the integrand is positive at every instant, as the kinematic risk is, or only during rare events.

At trajectory level the two families of measure are correlated in the expected direction but only moderately, and Table 12 shows that the tier system is externally valid: conflict rate rises monotonically across the tiers of both kinematic features, from 1.21% to 8.32% for risk intensity and from 1.37% to 10.60% for peak risk, and the conditional rate rises in the same way, with all four trends significant at p < 10^-20^. Trajectories in the Very High tier are substantially more likely to have been involved in a close interaction. The tiers therefore mean something in interaction terms at trajectory level, even though the indicator behind them does not register task complexity. Fig 8 shows the same gradient as a bar chart for both tier systems.

**Table 12:** Conflict rate by risk tier (external validation).

Tier system	Tier	n	Valid TTC	Conflicts (TTC < 3 s)	Conflict rate, overall	Conflict rate, conditional	Median TTC (s)
Risk intensity	Low	3,064	1,942	37	1.21%	1.91%	23.64
	Medium	6,128	4,331	123	2.01%	2.84%	16.82
	High	2,451	1,772	93	3.79%	5.25%	13.17
	Very High	613	469	51	8.32%	10.87%	10.59
Peak risk	Low	3,064	1,867	42	1.37%	2.25%	23.46
	Medium	6,128	4,266	111	1.81%	2.60%	17.44
	High	2,451	1,881	86	3.51%	4.57%	13.02
	Very High	613	500	65	10.60%	13.00%	9.17

**Fig 8:**
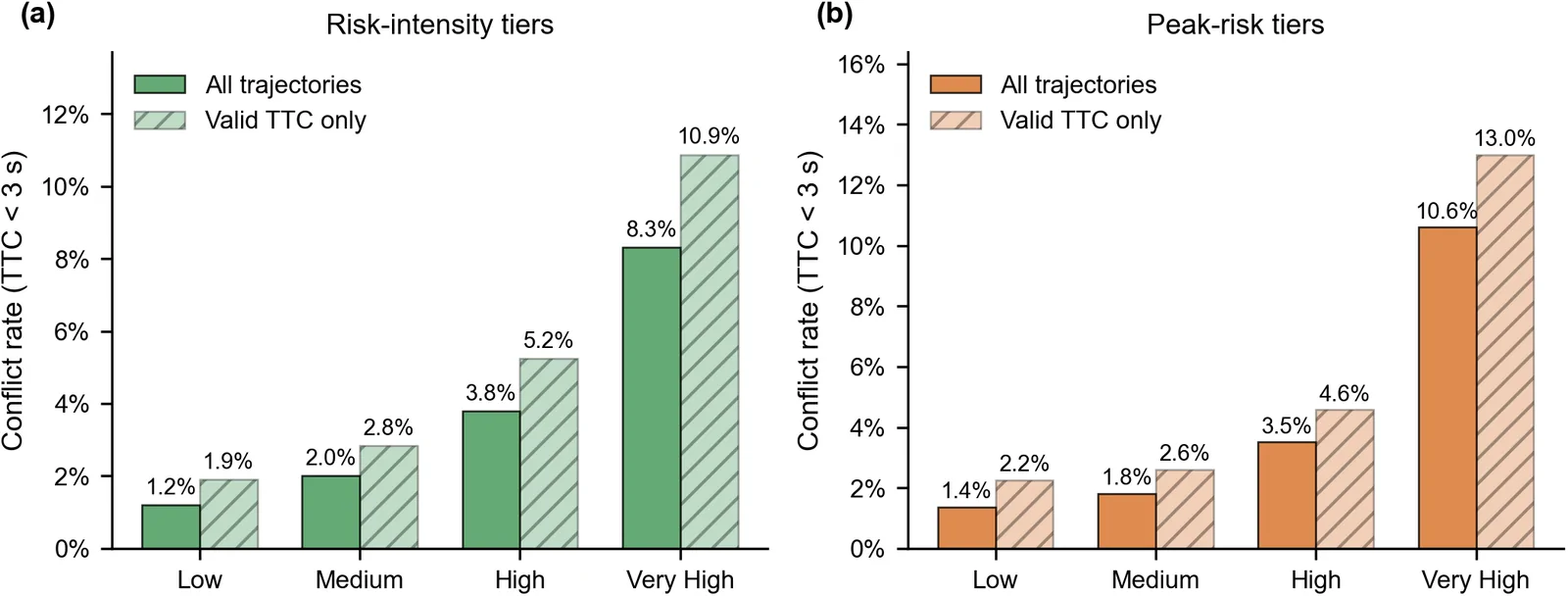
Conflict rate by risk tier for (a) risk-intensity tiers and (b) peak-risk tiers.

### 3.6 Sensitivity analyses

Five sensitivity analyses were conducted, and none changes any conclusion. Substituting equal weights for the entropy-derived weights leaves the vehicle-type effect at d = −0.247 against −0.244 and the length–intensity correlation at −0.659 against −0.767. Omitting the low-speed filter gives d = −0.233 and −0.767; omitting the Savitzky-Golay smoothing gives d = −0.249 and −0.767. Doubling the jerk constant to 5.0 m/s³, as some kinematic risk studies do, raises jerk saturation from 1.4% to 0.04% and gives d = −0.239 and −0.792. We keep the ISO-based value, which places the bound at the extreme tail of the observed jerk distribution rather than beyond it.

The effect of sampling rate was examined using the 29.97 Hz material available at two sections. Progressively decimating to 15, 10, 6 and 5 Hz, with the time step handled correctly at each rate, changes mean cumulative risk by 9.6% between the extremes and preserves every ranking (Table 13). Derivative-based indicators are therefore mildly rate-dependent, and comparisons across datasets with differing sampling rates should be made on a common resampled grid, as here.

**Table 13:** Sampling-rate sensitivity (sections 2 and 3, 29.97 Hz source).

Sampling rate	Mean jerk component	Mean cumulative risk	Relative to 30 Hz
29.97 Hz	0.159	1.813	1.000
14.99 Hz	0.133	1.708	0.942
9.99 Hz	0.126	1.680	0.927
5.99 Hz	0.120	1.651	0.910
5.00 Hz	0.117	1.639	0.904

How quality-rejected trajectories are handled also matters for the interaction measure. Excluding them from the traffic scene as well as from the analysis lowers conflict rates substantially — section 5 falls from 2.43% to 2.24% and the correlation between complexity and conflict rate from +0.905 to +0.880 — because removing a vehicle leaves the following vehicle with a more distant leader. Retaining them as potential leading vehicles, as we do, avoids this bias.

Residual diagnostics show that the raw-scale models depart from normality, with skewness of 1.59 and kurtosis of 9.93 for cumulative risk, and that residual variance differs significantly between sections for every outcome. A logarithmic transformation largely repairs the normality violation (skewness 0.32, kurtosis 0.71, Shapiro–Wilk W = 0.993 for cumulative risk) and leaves every conclusion unchanged: the length coefficient remains positive for cumulative risk (β = +0.241, p < 0.001), negative for risk intensity (β = −0.117, p = 0.004) and null for peak risk (β = +0.018, p = 0.549). Heteroscedasticity across sections persists on the log scale, a further reason to base inference on permutation. We report the raw scale, on which the indicator is defined; percentile-based tiers are in any case invariant under monotone transformation.

## 4. Discussion

At this sample size no configuration effect other than the arithmetic one is significant on either dimension, but the two dimensions respond differently. Task complexity — by construction a measure of how much merging and diverging activity a section must accommodate — is more strongly associated with conflict rate than with risk intensity (permutation p = 0.028), and no association with any kinematic outcome was detected (p = 0.915 for risk intensity). A study using kinematic indicators alone, as many do, would have concluded that complexity is irrelevant to weaving-section risk, and nothing in the kinematic data would have shown otherwise.

That the two families correlate imperfectly is already known; what these data add is that they differ systematically in which environmental features they register at all. A vehicle travelling smoothly at a short gap receives a low kinematic score, so a section that forces many such encounters looks unremarkable on kinematic indicators while generating conflicts at three times the rate of a simpler one. Kinematic screening, however convenient at scale, should not be used on its own to compare facilities.

The vehicle-type comparison makes the same point in a different way. Because every component is normalised by a constant, whether heavy vehicles appear safer or more dangerous is decided by the constants assumed: common constants make them safer by a quarter of a standard deviation, type-specific constants make them more dangerous by one standard deviation. The kinematic indicator cannot answer the question. The interaction measure can, because it involves no such normalisation, and it shows heavy vehicles involved in fewer close interactions (1.08% against 2.68% of trajectories below TTC = 3 s), which we read as more conservative manoeuvring. Neither measure speaks to crash severity, where vehicle mass matters a great deal.

A second methodological point concerns the choice of outcome. Cumulative risk, being a time integral of a quantity that is positive at every instant, grows with traversal time, and traversal time is determined largely by section length (r = 0.989 in these data). Comparing cumulative risk across sections of differing length therefore confounds a substantive effect with an arithmetic one, and can reverse the apparent direction of the length effect. This does not condemn every time-integrated measure: time spent in conflict is also an integral over time, but accumulates only during the rare intervals when a conflict is under way, and correlates with length at +0.456 rather than +0.948. What matters is whether the integrand is positive throughout the traversal.

The individual-level findings are consistent across both dimensions and carry more weight than any section-level result here, because they rest on 12,256 observations rather than seven. Merging and diverging movements are the most demanding on every kinematic measure and generate the highest conflict rates, which supports attention to deceleration-lane geometry, advance signing and lane discipline at diverging points. The entry-speed result is easily misread: higher entry speed goes with lower cumulative risk, but that reflects shorter exposure rather than safer driving, since risk intensity is 12.7% higher in the fastest tertile. Raising entry speeds would not reduce risk, only the time spent accumulating it. Entry speed also cannot be separated from prevailing traffic conditions here, since congestion produces both lower speeds and shorter headways.

Several limitations constrain these conclusions, and the most fundamental is the number of sections. Seven clusters provide little power, and the permutation tests make the consequence concrete: associations that appear decisive under parametric assumptions — weaving length on risk intensity at p = 0.009, task complexity on conflict rate at p = 0.001 — become inconclusive when the reference distribution is generated by reassigning configurations among the seven sections. The same arithmetic applies to much of the published weaving-section literature, where section counts are similar and permutation tests are not usually reported. Testing these relationships confirmatorily would need twenty sections or more.

Other limitations are narrower. The data come from one city, during morning peak periods and in clear weather, which limits generalisability. The interaction measure identifies leading vehicles by lateral proximity rather than lane membership and assumes a common vehicle length, and a valid time-to-collision exists for 69.5% of trajectories; on the conditional rate the association between task complexity and conflict is weaker (p = 0.166). The tracking-quality filter removes 1.5% of trajectories whose acceleration traces contain physically unattainable discontinuities; these were concentrated in the highest risk tiers, and keeping them would have inflated both the kinematic and the interaction measures. The risk tiers are defined by sample percentiles and validated against a surrogate measure, not against crash outcomes.

The obvious next step is a larger set of sections with systematic variation in length and complexity, which would allow the pattern proposed here to be tested confirmatorily. Linking both dimensions to crash records would let their relative contributions be weighted rather than reported side by side, and extending the interaction analysis to explicit lane-change conflict pairs, and to weather and time of day, would strengthen external validity.

## 5. Conclusions

This study analysed 12,256 UAV-derived vehicle trajectories from seven highway weaving sections along two dimensions, a kinematics-based composite risk indicator and an interaction-based conflict measure, with section-level inference assessed by exact permutation. Four conclusions follow.

First, cumulative risk is unsuitable for comparing weaving sections of differing length. Because Eq (10) integrates over the traversal, it is mechanically dominated by traversal time, which correlates with weaving length at r = 0.989; the time-averaged intensity of Eq (12) should be used instead.

Second, the two measurement dimensions differ in what they detect. Task complexity is more strongly associated with interaction-based conflict rate than with kinematic risk intensity (permutation p = 0.028), although with seven sections neither association is significant on its own. Kinematic indicators characterise the demand placed on the individual driver, and no association between them and the interaction burden a section imposes was detected in these data.

Third, individual behaviour dominates risk variation and both dimensions agree about it. Merging and diverging movements carry the highest risk and the highest conflict rates; higher entry speed shortens exposure while raising risk per unit time. The vehicle-type comparison is not identified by the kinematic indicator, whose sign follows from the normalising constants assumed; on the interaction measure heavy vehicles are involved in fewer close interactions than passenger cars.

Fourth, percentile-based risk tiers carry interaction-relevant meaning at the level of individual trajectories, with conflict rate rising monotonically from 1.2% to 8.3% across intensity tiers. This supports their use for screening, though not as absolute safety standards, which would need crash calibration.

For practice these results direct attention to merging and diverging movements, and caution against evaluating weaving sections either by accumulated exposure measures that reward brevity or by kinematic indicators alone.

## Supporting information

S1 Code Analysis code. The complete processing and analysis pipeline.
(ZIP)

S2 Data Processed trajectory dataset. Per-section 10 Hz resampled time series for all seven weaving sections.
(ZIP)
